# Essentialist and Nonessentialist Views of Cognitive Aging in Older Age: The Role of Working Memory, Cognitive Inhibition and Psychological Well-Being

**DOI:** 10.3390/jintelligence14100238

**Published:** 2026-10-02

**Authors:** Enrico Sella, Elena Carbone, Riccardo Domenicucci, Diletta Signori, Giorgio Arcara, Erika Borella

**Affiliations:** 1 Department of General Psychology, University of Padova, 35122 Padova, Italy; elena.carbone@unipd.it (E.C.); riccardo.domenicucci@unipd.it (R.D.); diletta.signori@phd.unipd.it (D.S.); giorgio.arcara@unipd.it (G.A.); erika.borella@unipd.it (E.B.); 2 IRCCS San Camillo Hospital, 30126 Venice, Italy

**Keywords:** working memory, cognitive inhibition, views of cognitive aging, essentialism of aging

## Abstract

Views of cognitive aging refer to how people think and believe about age-related cognitive changes with aging, including whether they perceive these age-related cognitive changes as fixed (essentialism of cognitive aging) or as potentially malleable (nonessentialism). This cross-sectional study examined the role of cognitive functioning—in particular working memory and cognitive inhibition/interference performance—and psychological well-being in explaining views of cognitive aging among older adults. A total of 237 community-dwelling older adults aged 64–85 years completed the Views of Cognitive Aging questionnaire, along with verbal working memory, cognitive inhibition/interference tasks, and a Psychological Well-Being questionnaire. Regression analyses showed that cognitive performance and psychological well-being explained a significant, though modest, part of the variance mainly in nonessentialist (malleable) views of cognitive aging. Better working memory performance was associated only with stronger generalized and personal nonessentialist views. In addition, greater cognitive resistance to proactive interference, but not inhibition, and psychological well-being were positively associated with generalized nonessentialist views of cognitive aging. These associations were more evident among adults aged 75–85 years than among those aged 64–74 years. Overall, these findings suggest that cognitive performance and psychological well-being contribute differently to the views of age-related cognitive changes and are more closely related to flexible, rather than fixed, views of cognitive aging, particularly in adults 75 and older. The examination of views of cognitive aging could be included in assessment protocols aimed at promoting healthy aging.

## 1. Introduction

Subjective views of aging (VoA) refer to individuals’ beliefs, experiences, and expectations of older adults as a social group and their own aging process (Shrira et al., 2022). Within this broad conceptual framework, generalized VoA include old age stereotypes and, more recently, (non)essentialist beliefs about aging, which have attracted increasing attention because they capture the extent to which aging is perceived as fixed and biologically determined rather than as a malleable process (Weiss, 2018). Personal VoA, in turn, encompasses subjective or felt age (Montepare, 2009), Attitudes Toward one’s Own Aging (ATOA; Lawton, 1975) and awareness of age-related changes (AARC; Diehl et al., 2014). Generalized and personal VoA have been associated with a wide range of outcomes related to physical and mental health, quality of life, and longevity (Carbone et al., 2026; Sella et al., 2025; Westerhof et al., 2023).

An emerging body of research has also examined how both cognitive functioning and well-being are related to VoA in midlife and older adulthood (Fernández-Ballbé et al., 2023; Carbone et al., 2025; Sella et al., 2026).

Regarding cognition, at the level of generalized VoA, correlational and longitudinal studies have shown that more negative beliefs/stereotypes about aging are associated with poorer episodic memory performance and an increased risk of cognitive decline or dementia (B. Levy, 1996; B. R. Levy et al., 2018). More recently, essentialist beliefs about aging have also been linked to memory performance: when measured along a continuum from aging as relatively immutable and unchangeable (essentialist) to relatively flexible and modifiable (nonessentialist) process, stronger essentialist beliefs have been associated with poorer episodic memory performance (Weiss & Weiss, 2016; Weiss, 2018). However, this few evidence has mainly focused on essentialist beliefs and memory outcomes. Initial evidence has also linked nonessentialist beliefs about cognitive aging to cognitive performance, including working memory (WM) and attentional measures (Sella et al., 2026). However, the role of cognitive abilities in influencing essentialist and nonessentialist views of age-related specifically in cognitive changes among older adults remains insufficiently understood.

Associations between personal VoA and cognitive outcomes have been more consistently documented (Fernández-Ballbé et al., 2023, for a review). Much of the empirical evidence comes from studies that examine associations between felt age or ATOA and cognitive outcomes, particularly global cognition and episodic memory performance. Studies using AARC found AARC-Gains and AARC-Losses to be—positively and negatively, respectively—related to verbal reasoning and verbal short-term memory (Sabatini et al., 2023), whereas Carbone et al. (2025), found that both greater AARC-Gains and AARC-Losses to be—positively and negatively, respectively—associated with verbal WM performance, whereas felt age and ATOA showed negligible effects.

Notably, to date, although most evidence has examined VoA as antecedents or predictors of cognitive performance (e.g., Carbone et al., 2025; Sabatini et al., 2023), the opposite direction remains much less explored. Few studies examining whether cognitive functioning predicts VoA have mainly focused on personal VoA dimensions, and the findings appear to differ depending on the specific dimension considered.

Notably, global cognitive functioning (as measured with the Mini-Mental State Examination, Folstein et al., 1975), was not a significant predictor of felt age in community-dwelling older adults aged 65 to 90 years (Schönstein et al., 2021), whereas it significantly predicted ATOA in older adults aged 60 to 79 years (Wang et al., 2020). More recent longitudinal evidence further showed that better global cognitive functioning (as measured by Mini-Mental State Examination), was associated with a more positive ATOA over time in community-dwelling older adults aged 60 years and older (Mei et al., 2026).

When different cognitive domains are considered, the evidence is even more limited. Hajek et al. (2020) found that the longitudinal change in processing speed (assessed with the Digit Symbol Test) positively predicted ATOA in adults aged 40 and older. Regarding AARC, Sabatini et al. (2021) found that only long-term negative changes in processing speed and perceptual reasoning predicted lower AARC-Gains, while changes in other cognitive domains, including short-term memory and verbal reasoning, were not significantly associated with AARC-Losses.

Nonetheless, these few studies have focused mainly on broad personal VoA dimensions, i.e., felt age, ATOA, or behavior-specific i.e., AARC, whereas the role of cognitive abilities in essentialist and nonessentialist VoA specifically related to age-related cognitive changes has not yet been examined. Recently, we developed the Views of Cognitive Aging questionnaire (VoCA; Sella et al., 2026), a novel measure that assesses both generalized and personal views of age-related cognitive changes with aging and distinguishes, within each domain, between essentialist and nonessentialist beliefs about cognitive aging.

Such a distinction aligns with theoretical accounts of the VoA-health nexus. Essentialist beliefs about cognitive aging assume that age-related cognitive changes are fixed, inevitable, and less open to individual agency, thus implying lower perceived efficacy. Conversely, nonessentialist beliefs frame aging as malleable, they may reinforce individuals’ beliefs in their capacity to actively shape the aging process (Diehl et al., 2023; Weiss & Diehl, 2021), and support cognitive functioning through effort, engagement, and health-related lifestyles or choices. VoCA also reflect the broader perception that age-related cognitive changes are modifiable and manageable through controllable factors—such as lifestyle at a general level or choices at a personal level-, in the case of nonessentialist beliefs or largely beyond individual control and more inevitable, in the case of essentialist beliefs about cognitive aging. Essentialist and nonessentialist VoCA are thus theoretically related, at least to some extent, to self-efficacy—defined as individuals’ beliefs in their ability to organize and execute the actions required to attain desired outcomes (Bandura & Wessels, 1997)—as well as to control beliefs and locus of control—which refer to the extent to which individuals perceive outcomes as controllable and dependent on internal or external factors (e.g., Lachman, 2006). However, it is important to note that the VoCA go beyond assessing how capable individuals feel of acting to maintain or support their cognitive functioning and changes with aging, or whether they attribute cognitive outcomes to internal or external causes. Instead, essentialist and nonessentialist VoCA refer and capture more specifically to how individuals construe—across the lifespan—cognitive aging itself, whether age-related cognitive changes are viewed and perceived as fixed and uncontrollable or as a malleable and modifiable process open to change (Sella et al., 2026).

Therefore, one of the aims of this study was to examine whether and to what extent key cognitive abilities in aging—such as WM and inhibitory processes—(Craik & Salthouse, 2011) contribute to generalized and personal VoCA may help clarify differences between essentialist and nonessentialist beliefs about cognitive aging in older adulthood. Verbal WM is relevant to the active maintenance and processing of verbal information to be used in complex tasks related to everyday functioning, and is one of the cognitive mechanisms most sensitive to aging (Borella et al., 2011, 2008). However, the relationship between WM and VoA has only rarely been examined (Sabatini et al., 2023), particularly with complex WM tasks—as this used in the present study-, despite some evidence linking complex WM to personal VoA (Carbone et al., 2025).

Cognitive inhibition and interference-related processes are, then, among the main cognitive mechanisms underlying age-related cognitive decline (e.g., Borella et al., 2008, 2011; Hasher et al., 2007), or age-related changes in other crucial mechanisms such as—verbal—WM (Borella et al., 2011). They may therefore also be relevant to how older adults interpret age-related changes in their cognitive functioning.

Considering these constructs together made it possible to examine whether different, though related, cognitive mechanisms were associated with generalized and personal VoCA, framed as essentialist or, conversely, nonessentialist views of age-related cognitive changes with aging. Yet there are both conceptual and empirical reasons to expect that cognitive functioning may also be associated with how older adults perceive cognitive aging. From a conceptual perspective, Diehl et al. (2014) proposed that cognitive abilities should be considered not only as outcomes of VoA, but also as psychological resources involved in how individuals interpret the aging process (including cognitive one).

VoA has traditionally focused also on outcomes closely related to the broader concept of well-being (e.g., Sabatini et al., 2021; Westerhof et al., 2023), and psychological well-being (PWB) in relation to VoA is understudied in older age.

For generalized VoA, Weiss (2018) found that essentialist beliefs about aging are indirectly associated with increased stress reactivity, which is considered a proxy of lower PWB, although the effects are modest. Only one study examined essentialist beliefs and PWB. Prati (2024) assessed essentialist beliefs about cognitive aging (measured with three-item scale from the Personality in Intellectual Aging Contexts Scale; Lachman et al., 1982) in relation to PWB (using a version of Ryff’s scale; Boylan & Ryff, 2015) in a large sample of adults aged 18 to 84 years, and found that stronger essentialist beliefs about cognitive aging were associated with poorer PWB; moreover, latent change score models showed that higher essentialism predicted poorer PWB over time.

In addition, the only two studies linking personal VoA to PWB reported mixed results. Brothers et al. (2017) showed that AARC-Gains were positively associated with PWB and AARC-Losses were negatively associated with PWB in individuals aged 40 to 98 years, whereas Nosrati et al. (2026) found positive correlations between PWB and global self-perceptions of aging (assessed with Aging Perceptions Questionnaire) and AARC-Gains and AARC-Losses, while attitudes toward aging were not significantly associated with PWB.

However, whether and how PWB may relate to essentialist and nonessentialist beliefs about cognitive aging—at generalized and personal level—remains unexplored. Examining such an association, together with cognitive abilities, could help clarify individual differences in essentialist and nonessentialist views of age-related cognitive changes, an issue that remains worthy of further investigation.

Hence, the present cross-sectional study investigated the role of core cognitive abilities and PWB in explaining essentialist and nonessentialist VoCA among community-dwelling older adults. By distinguishing between essentialist and nonessentialist beliefs about cognitive aging, the study extended previous research on VoA by examining whether these two theoretically distinct views of age-related cognitive changes could be differentially predicted by both cognitive functioning and PWB in older adults. Essentialist and nonessentialist beliefs about cognitive aging, from both generalized and personal perspectives, were operationalized and examined using the novel VoCA questionnaire (Sella et al., 2026). Complex WM and cognitive inhibitory tasks, classically used in aging studies, were thus selected. In particular, the Listening Span Test allowed us to assess both—verbal—WM performance and susceptibility to proactive interference, indexed by intrusion errors (see Borella et al., 2011, 2008), whereas the Hayling Sentence Completion Test allowed to assess controlled or executive attention in older adults (Borella et al., 2011; Palombo & Foran, 2025). PWB was measured using the “BEN-SSC” Psychological Well-Being Questionnaire, which provides a global index of psychological—eudaimonic—well-being (De Beni et al., 2008).

Previous studies have linked cognitive and memory performance to essentialist beliefs about aging (Weiss & Weiss, 2016; Weiss, 2018), although with small effect sizes. Given that VoCA are theoretically grounded in essentialist beliefs about aging (Sella et al., 2026), we expected better verbal WM and inhibitory performance would be associated with lower generalized essentialist VoCA. Moreover, in line with the only study that has assessed nonessentialist beliefs about cognitive aging in relation to cognitive performance (including WM; Sella et al., 2026), we expected better verbal WM and inhibitory performance to be associated with higher generalized nonessentialist VoCA, although these effects were expected to be small but significant.

As for personal VoCA, similarly, in line with previous findings showing positive associations between cognitive functioning (encompassing also WM) and personal VoA dimensions (e.g., Mei et al., 2026; Sabatini et al., 2023; Voelkner & Caskie, 2023), we hypothesized that better verbal WM performance would be associated with lower personal essentialist VoCA and higher personal nonessentialist VoCA, albeit with small effect sizes. Furthermore, given that better global cognitive/memory functioning has been linked, although modestly, to various VoA constructs (e.g., Weiss, 2018; Carbone et al., 2025) and considering the relationship between verbal WM and inhibition (e.g., Borella et al., 2011; de Ribaupierre, 2001), we expected that better inhibitory functioning would be associated with lower essentialist VoCA and higher nonessentialist VoCA at both the generalized and personal levels. Given the novelty of this question in the VoA literature, these hypotheses were formulated with caution.

Regarding PWB, in line with previous evidence on generalized and personal VoA (Brothers et al., 2017; Prati, 2024; Nosrati et al., 2026), we expected higher PWB to show small-to-moderate associations with both generalized and personal VoCA. Specifically, a higher PWB was expected to be associated with a lower generalized and personal essentialist VoCA and with a higher generalized and personal nonessentialist VoCA.

In addition, because subjective experiences of aging and the relation between cognition and aging-related perceptions can differ between older adults due to substantial interindividual differences in aging, we explored whether the pattern of associations between verbal WM, inhibitory processes, PWB, and VoCA differed between young-old and old-old adults.

Finally, control variables were considered in our models (see below) because they are known to be associated with—verbal—WM and cognitive inhibition in older age: age and gender have been associated with—verbal—WM and inhibitory processes (Borella et al., 2008; Siegel & Eich, 2021), while education is commonly used as a proxy of cognitive reserve and has been linked to older adults’ cognitive performance (Pliatsikas et al., 2019). Given their role also in PWB in older adults (Ryff & Keyes, 1995; Boylan et al., 2025), these variables were controlled for when examining the contribution of cognitive functioning and PWB to VoCA.

## 2. Materials and Methods

### 2.1. Participants

A convenience sample of 237 healthy older adults (56% women), aged between 64 and 85 years old. Data were collected between February 2025 and March 2026. All participants were native Italian speakers residing in Italy. Eligibility criteria included good cognitive functioning, defined as a score above the cutoff of 22 on the Montreal Cognitive Assessment (corresponding to an equivalent score of 2; see Dapor et al., 2025), as well as good physical and mental health, as assessed through a semi-structured interview (De Beni et al., 2008). Participants were drawn from a broader project aimed at validating a cognitive and psychological assessment protocol developed by the Unit of Psychology of Aging from the local University and were recruited on a voluntary basis through the experimenters’ networks and the collaboration with local associations or recreational centers for healthy adults and older adults. Descriptive information on age, gender, education and retirement status is reported in Table 1. For the purposes of the present study, all participants who had completed the measures of interest were included in the analyses. The study was conducted in accordance with the Declaration of Helsinki and was approved by the local Ethics Committee (protocol no. 996-b).

### 2.2. Materials

#### 2.2.1. Essentialist and Nonessentialist Views of Cognitive Aging

The VoCA (Sella et al., 2026) is a 15-item questionnaire developed to assess essentialist and nonessentialist beliefs about cognitive aging. The questionnaire includes a generalized section (VoCA-G), which captures beliefs about cognitive aging in general, and a personal section (VoCA-P), which assesses beliefs about one’s own cognitive aging. In VoCA-G, nonessentialist beliefs reflect the view that cognitive aging is malleable (e.g., “With aging, memory can be improved by staying actively engaged”), whereas essentialist beliefs reflect the view that cognitive aging is fixed and inevitable (e.g., “Memory ability worsens with aging”). In VoCA-P, nonessentialist beliefs reflect a sense of agency over one’s own cognitive functioning (e.g., “When I make an effort, I notice that I am able to use my mental abilities despite getting older”), whereas essentialist beliefs reflect a perceived lack of control (e.g., “Getting older does not allow me to improve my mental abilities”). Four total scores were computed: generalized nonessentialism, generalized essentialism, personal nonessentialism, and personal essentialism. These scores were used as variables in the analyses. In the present sample, the four VoCA indices showed acceptable-to-good internal reliability: generalized nonessentialism (VoCA-GnE; ω = 0.71), generalized essentialism (VoCA-GE; ω = 0.74), personal nonessentialism (VoCA-PnE; ω = 0.67), and personal essentialism (VoCA-PE; ω = 0.81).

#### 2.2.2. Cognitive Tasks

***Verbal WM.*** The Listening Span Test (LST; Borella et al., 2008) consists of sets containing increasing numbers (from two to six) of simple sentences, organized into two parts. In total, 40 sentences are presented, with a 1.5-s interval between consecutive sentences. Participants are required to listen to each sentence, judge its plausibility by indicating whether it is true or false, and retain the final word. At the end of each set, they are asked to recall the last word of each sentence in the correct order of presentation. Two variables were derived from the task: WM performance (indexed by the number of correctly recalled final words; i.e., LST words recalled) and resistance to proactive interference (PI; the proportion of intrusion errors relative to the number of correctly recalled words; i.e., LST intrusion index). Cronbach’s α for the LST words recalled score was 0.75 (see De Beni et al., 2008). Reliability was not estimated for the LST intrusion index, as this variable is a derived proportional index of proactive interference rather than a multi-item performance score.

***Cognitive inhibition.*** The Hayling Sentence Completion Test (Hayling test; Borella et al., 2008) measures the ability to inhibit dominant verbal responses. Participants are presented with high-cloze sentences missing the final word and are asked to complete them with an expected word (initiation condition) and with a word that is unrelated in meaning but grammatically appropriate (inhibition condition). The task includes 28 sentences, with 14 sentences administered in each condition. The dependent variable consists of the inhibition index, calculated as the sum of the errors committed (Borella et al., 2008), with higher scores reflecting greater difficulty in suppressing dominant responses. Cronbach’s α for the Hayling inhibition score was 0.70.

#### 2.2.3. PWB Questionnaire (“BEN-SSC–Questionario Ben-Essere Psicologico”; De Beni et al., 2008)

PWB was assessed using the BEN-SSC (De Beni et al., 2008), a 37-item questionnaire developed for the assessment of PWB in adults and older adults in Italy. The instrument is rooted in the eudaimonic perspective of PWB and includes three main dimensions: personal satisfaction, coping strategies, and emotional competence. Participants rate each item on a 4-point Likert scale ranging from 1 (never) to 4 (always), and a total score can be computed by summing responses across items. In the present study, the scale showed good internal reliability (ω = 0.90), supporting the use of a global score in the analyses.

### 2.3. Procedure

Each participant provided written informed consent and completed the study measures during a single individual assessment session (90 min) administered by a trained psychologist using a fixed order. All participants first completed the demographic questionnaire, followed by the LST and three additional brief cognitive tasks from the broader healthy-aging assessment protocol, which together lasted approximately 6 min and were not analysed in the present study. Participants then completed the Hayling test, the Montreal Cognitive Assessment, the BEN-SSC, and the VoCA. Between tests, the experimenter checked whether participants were experiencing fatigue and, if necessary, allowed a few minutes of rest. None of the participants required or asked for a break.

### 2.4. Statistical Analyses

First, Pearson’s correlation coefficients were calculated between all study variables of interest (Table 2). Given the number of correlations tested, *p*-values were adjusted for multiple comparisons using the Benjamini-Hochberg false discovery rate procedure (Benjamini & Hochberg, 1995).

Then, to examine the variance explained in essentialist and nonessentialist VoCA, a series of hierarchical regression models was conducted using the four VoCA dimensions as dependent variables (i.e., generalized nonessentialism [VoCA-GnE] and essentialism [VoCA-GE]; personal nonessentialism [VoCA-PnE] and essentialism [VoCA-PE]). In Step 1, sociodemographic variables (age, gender, and education) were entered as control variables. In Step 2, cognitive variables were simultaneously added, including verbal WM (LST words recalled), and cognitive inhibition indices (LST intrusion index—resistance to PI-, and the Hayling test—controlled cognitive inhibition-). This step was designed to estimate the unique associations between cognitive functioning and VoCA while accounting for relevant sociodemographic differences. In Step 3, PWB was added to evaluate whether these associations remained after accounting for psychological well-being or whether PWB display an incremental contribution to the explained variance beyond sociodemographic and cognitive variables. These models were conducted first in the total sample and then separately in the young-old (64–74 years) and old-old (74–85 years) subsamples (Supplementary Materials, PART S2).

All analyses were performed using R statistical software (version 4.3.2, R Core Team, 2026).

## 3. Results

### 3.1. Correlations in the Full Sample

Table 2 shows correlations of interest with significance levels adjusted using the Benjamini-Hochberg FDR procedure in the total sample and by age group. In the total sample, generalized nonessentialist views of cognitive aging (VoCA-GnE) were positively associated with verbal WM. Considering personal VoCA, personal nonessentialist views (VoCA-PnE) were positively associated with verbal WM performance. In contrast, generalized essentialist (VoCA-GE) and personal essentialist (VoCA-PE) views were not significantly associated with any cognitive measures.

Considering covariates, age was negatively associated with VoCA-PnE, whereas gender was negatively associated with VoCA-PE. Education was not significantly associated with any VoCA dimension. PWB was positively associated with VoCA-GnE and VoCA-PnE, and was not significantly associated with either generalized or personal essentialist VoCA dimensions.

In the young-old group, neither generalized nor personal VoCA dimensions were significantly associated with cognitive measures or PWB. Among sociodemographic variables, only gender (being men) was negatively associated with VoCA-PE. In the old-old group, VoCA-GnE was positively associated with verbal WM performance and PWB, whereas VoCA-PnE was positively associated with PWB. No significant associations emerged between essentialist VoCA dimensions and cognitive measures or PWB, and none of the sociodemographic variables was significantly associated with any VoCA dimension.

### 3.2. Hierarchical Regressions in the Full Sample

**Generalized VoCA.** For VoCA-GnE, the final model explained 16.4% of the variance (Table 3; Figure 1). In Step 1, sociodemographic variables explained 3.5% of the variance, with age emerging as the only significant negative predictor, (β = −0.16, 95% CI [−0.29, −0.02], *p* = .014). In Step 2, cognitive variables explained an additional 6.5% of the variance, for a total of 10.0%, with verbal WM positively predicting VoCA-GnE (β = 0.27, 95% CI [0.12, 0.42], *p* < .001) and LST intrusion index also emerging as a significant positive predictor (β = 0.14, 95% CI [0.00, 0.28], *p* = .047). In Step 3, adding PWB explained an additional 6.4% of the variance (ΔR^2^ = 0.064). In the final model, verbal WM (β = 0.28, 95% CI [0.14, 0.42], *p* < .001), LST intrusion index (β = 0.15, 95% CI [0.02, 0.28], *p* = .027), and PWB (β = 0.25, 95% CI [0.13, 0.37], *p* < .001) were significant positive predictors.

For VoCA-GE, the model was not significant at Step 1. In Step 2, cognitive variables explained an additional 2.6% of the variance, for a total of 4.9%, and the overall model was marginally significant (*p* = .070), with verbal WM emerging as the only significant positive predictor (β = 0.17, 95% CI [0.02, 0.32], *p* = .029). In Step 3, PWB explained an additional 1.0% of the variance (ΔR^2^ = 0.010). In the final model, verbal WM remained the only significant predictor (β = 0.18, 95% CI [0.03, 0.34], *p* = .018). The final model remained marginally significant (*p* = .050; 5.9% of the variance; Table 3; Figure 1).

**Table 2:** Pearson correlations among sociodemographic, cognitive, psychological, and views of cognitive aging variables in the total sample and by age group (young-old and old-old groups).

Variable	Sample	Age	1	2	3	4	5	6	7	8	9
Gender (1)	Full sample	−0.041	-								
	Young-old group	−0.079	-								
	Old-old group	0.049	-								
Education (2)	Full sample	−0.207 **	−0.168 *	-							
	Young-old group	−0.081	−0.111	-							
	Old-old group	−0.081	−0.222 *	-							
VoCA-GE (3)	Full sample	0.003	−0.139	0.083	-						
	Young-old group	0.085	−0.213	0.192	-						
	Old-old group	−0.089	−0.103	0.035	-						
VoCA-GnE (4)	Full sample	−0.084	−0.030	0.078	0.549 ***	-					
	Young-old group	0.068	−0.031	0.057	0.467 ***	-					
	Old-old group	−0.161	−0.034	0.077	0.585 ***	-					
VoCA-PE (5)	Full sample	0.035	−0.168 *	0.047	0.698 ***	0.388 ***	-				
	Young-old group	0.094	−0.334 *	0.173	0.637 ***	0.315 *	-				
	Old-old group	−0.083	−0.083	−0.002	0.725 ***	0.425 ***	-				
VoCA-PnE (6)	Full sample	−0.170 *	−0.080	0.083	0.624 ***	0.793 ***	0.414 ***	-			
	Young-old group	−0.130	−0.117	0.043	0.601 ***	0.726 ***	0.345 *	-			
	Old-old group	−0.115	−0.074	0.068	0.644 ***	0.821 ***	0.458 ***	-			
LST words recalled (7)	Full sample	−0.304 ***	−0.043	0.382 ***	0.100	0.197 **	0.000	0.183 *	-		
	Young-old group	−0.121	−0.015	0.489 ***	0.204	0.142	−0.006	0.170	-		
	Old-old group	−0.234 *	−0.080	0.268 **	0.061	0.217 *	0.024	0.154	-		
LST intrusion index (8)	Full sample	0.205 **	0.145	−0.260 ***	−0.117	−0.068	−0.074	−0.125	−0.453 ***	-	
	Young-old group	−0.031	0.083	−0.277	−0.128	0.089	−0.056	0.108	−0.378 **	-	
	Old-old group	0.181	0.186	−0.225 *	−0.121	−0.109	−0.094	−0.169	−0.462 ***	-	
Hayling test (9)	Full sample	−0.309 ***	0.051	0.274 ***	0.075	0.133	0.100	0.114	0.265 ***	−0.152	-
	Young-old group	0.000	0.158	0.254	0.090	0.199	0.117	0.049	0.222	−0.017	-
	Old-old group	−0.044	−0.037	0.206 *	0.087	0.086	0.133	0.089	0.182	−0.137	-
PWB (10)	Full sample	0.116	−0.070	−0.018	0.092	0.250 ***	−0.051	0.209 **	0.006	−0.029	−0.157 *
	Young-old group	0.103	0.031	0.063	0.180	0.171	−0.021	0.113	0.072	0.106	−0.132
	Old-old group	−0.057	−0.125	−0.030	0.042	0.311 ***	−0.081	0.293 **	0.018	−0.122	−0.111

Note. VoCA = Views of Cognitive Aging; GnE = Generalized nonessentialism; GE = Generalized essentialism; PnE = Personal nonessentialism; PE = Personal essentialism; LST = Listening Span test; PWB = Psychological Well-Being. Asterisks refer to FDR-corrected *p*-values: * *p* < .05, ** *p* < .01, *** *p* < .001.

**Table 3:** Hierarchical Regression Analyses Predicting Generalized Nonessentialist and Essentialist VoCA (-GnE and -GE) in the Full Sample.

	VoCA-GnE									VoCA-GE								
	*Step 1*			*Step 2*			*Step 3*			*Step 1*			*Step 2*			*Step 3*		
	*β*	*95% CI*	*p*	*β*	*95% CI*	*p*	*β*	*95% CI*	*p*	*β*	*95% CI*	*p*	*β*	*95% CI*	*p*	*β*	*95% CI*	*p*
Intercept	−0.00	−0.13–0.13	<.001	−0.00	−0.12–0.12	.023	−0.00	−0.12–0.12	.830	−0.00	−0.13–0.13	<.001	−0.00	−0.13–0.13	.030	0.00	−0.13–0.13	.264
Age	−0.16	−0.29–−0.03	.014	−0.09	−0.23–0.04	.189	−0.09	−0.22–0.04	.170	−0.09	−0.22–0.04	.165	−0.04	−0.18–0.10	.574	−0.03	−0.17–0.11	.684
Gender	0.01	−0.12–0.14	.897	−0.02	−0.15–0.10	.701	−0.00	−0.13–0.12	.948	−0.08	−0.21–0.05	.235	−0.10	−0.23–0.03	.148	−0.08	−0.22–0.05	.205
Education	0.06	−0.07–0.19	.362	−0.02	−0.16–0.12	.766	−0.03	−0.17–0.10	.616	0.07	−0.07–0.20	.329	0.01	−0.14–0.15	.931	−0.01	−0.15–0.14	.932
LST words recalled	-	-	-	0.27	0.12–0.42	<.001	0.28	0.14–0.42	<.001	-	-	-	0.17	0.02–0.32	.029	0.18	0.03–0.34	.018
LST intrusion index	-	-	-	0.14	0.00–0.28	.047	0.15	0.02–0.28	.027	-	-	-	0.06	−0.08–0.20	.396	0.07	−0.08–0.21	.355
Hayling test	-	-	-	0.09	−0.04–0.23	.174	0.13	−0.00–0.26	.059	-	-	-	0.07	−0.07–0.21	.302	0.09	−0.05–0.22	.228
PWB	-	-	-	-	-	-	0.25	0.13–0.37	<.001	-	-	-	-	-	-	0.10	−0.03–0.23	.130
R^2^/adjusted R^2^	0.035/0.022			0.100/0.076			0.164/0.138			0.023/0.010			0.049/0.024			0.059/0.030		
ΔR^2^/adjusted R^2^	-			0.065/0.054			0.064/0.062			-			0.026/0.014			0.010/0.006		
F	2.791			4.245			6.379			1.805			1.977			2.046		
*p*	.041			<.001			<.001			.147			.070			.050		

Note. VoCA = Views of Cognitive Aging; GnE = Generalized nonessentialism; GE = Generalized essentialism; LST = Listening Span test; PWB = Psychological Well-Being.

**Personal VoCA.** For VoCA-PnE, the final model explained 14.9% of the variance (Table 4; Figure 1). In Step 1, sociodemographic variables explained 6.5% of the variance, with age emerging as the only significant negative predictor (β = −0.24, 95% CI [−0.37, −0.11], *p* < .001). In Step 2, cognitive variables explained an additional 4.0% of the variance, for a total of 10.5%, with age negatively predicting VoCA-PnE (β = −0.18, 95% CI [−0.32, −0.05], *p* = .008) and verbal WM positively predicting VoCA-PnE (β = 0.22, 95% CI [0.07, 0.37], *p* = .003). In Step 3, adding PWB explained an additional 4.4% of the variance (ΔR^2^ = 0.044). In the final model, age remained a significant negative predictor (β = −0.18, 95% CI [−0.32, −0.05], *p* = .007), whereas verbal WM (β = 0.23, 95% CI [0.09, 0.38], *p* = .002) and PWB (β = 0.21, 95% CI [0.09, 0.33], *p* = .001) were significant positive predictors.

For VoCA-PE, the final model explained 3.8% of the variance (Table 4; Figure 1). In Step 1, sociodemographic variables explained 2.0% of the variance, but the model was not significant with any significant predictors. In Step 2, cognitive variables explained an additional 1.9% of variance, for a total of 3.9%, with gender emerging as the only significant predictor (β = −0.13, 95% CI [−0.27, −0.00], *p* = .047). In Step 3, PWB did not explain a significant additional part of variance in the VoCA-PE (ΔR^2^ = −0.001).

### 3.3. Hierarchical Regressions by Age Group

#### 3.3.1. Young-Old Group

**Generalized VoCA.** For VoCA-GnE, the final model explained 11.1% of the variance in the young-old group (Table S1; Figure S1). In Step 1, sociodemographic variables explained 0.9% of the variance, and the model was not statistically significant. In Step 2, cognitive variables explained an additional 7.4% of the variance, for a total of 8.3%, but the overall model was not significant. In Step 3, adding PWB explained an additional 2.8% of the variance (ΔR^2^ = 0.028). However, the final model was also not significant, and no significant predictors emerged.

For VoCA-GE, the final model explained 13.9% of the variance in the young-old group (Table S1; Figure S1). In Step 1, sociodemographic variables explained 8.1% of the variance, although the model was not statistically significant. In Step 2, cognitive variables explained an additional 2.5% of the variance, for a total of 10.6%, but the overall model was not significant. In Step 3, PWB explained an additional 3.3% of the variance (ΔR^2^ = 0.033). However, the final model remained non-significant, and no predictor reached statistical significance, although gender showed a marginal negative association with VoCA-GE (β = −0.22, 95% CI [−0.44, 0.00], *p* = .054).

**Personal VoCA.** For VoCA-PnE, the final model explained 10.5% of the variance in the young-old group (Table S2; Figure S1). In Step 1, sociodemographic variables explained 3.4% of the variance, and the model was not statistically significant. In Step 2, cognitive variables explained an additional 6.1% of the variance for a total of 9.5%, but the overall model was not significant. In Step 3, adding PWB explained an additional 1.0% of the variance (ΔR^2^ = 0.010); no significant predictors emerged.

For VoCA-PE, the final model was marginally significant (*p* = .05) and explained 16.6% of the variance in the young-old group (Table S2; Figure S1). In Step 1, sociodemographic variables explained 13.7% of the variance, and gender (being male) emerged as a significant negative predictor (β = −0.31, 95% CI [−0.52, −0.10], *p* = .004). In Step 2, sociodemographic and cognitive variables explained 16.6% of the variance, and the overall model was significant. Gender emerged as a significant negative predictor (β = −0.34, 95% CI [−0.55, −0.12], *p* = .003). In Step 3, PWB did not explain any additional variance in the outcome (ΔR^2^ = 0.000). In the final model, gender remained the only significant predictor (β = −0.34, 95% CI [−0.55, −0.12], *p* = .003).

#### 3.3.2. Old-Old Group

**Generalized VoCA.** For VoCA-GnE, the final model explained 21.4% of the variance in the old-old group (Table S3; Figure S2). In Step 1, sociodemographic variables explained 5.7% of the variance, with age emerging as the only significant negative predictor, (β = −0.22, 95% CI [−0.38, −0.07], *p* = .006). In Step 2, cognitive variables explained an additional 7.1% of the variance, for a total of 12.8%, with age emerging as a significant negative predictor (β = −0.18, 95% CI [−0.33, −0.02], *p* = .027) and verbal WM as a significant positive predictor (β = 0.28, 95% CI [0.11, 0.45], *p* = .002). In Step 3, adding PWB explained an additional 8.6% of the variance (ΔR^2^ = 0.086). In the final model, verbal WM (β = 0.31, 95% CI [0.15, 0.48], *p* < .001), LST intrusion index (β = 0.19, 95% CI [0.02, 0.35], *p* = .026), and PWB (β = 0.29, 95% CI [0.14, 0.44], *p* < .001) were significant positive predictors.

For VoCA-GE, the final model explained 6.1% of the variance in the old-old group (Table S3; Figure S2). In Step 1, sociodemographic variables explained 3.2% of the variance, and the model was not statistically significant, although age emerged as a significant negative predictor (β = −0.17, 95% CI [−0.33, −0.01], *p* = .038). In Step 2, cognitive variables explained an additional 2.6% of the variance, for a total of 5.8%, but the overall model was not significant. In Step 3, PWB explained an additional 0.3% of the variance (ΔR^2^ = 0.003).

**Personal VoCA.** For VoCA-PnE, the final model explained 15.5% of the variance in the old-old group (Table S4; Figure S2). In Step 1, sociodemographic variables explained 4.1% of the variance, and the model was not statistically significant, although age emerged as a significant negative predictor (β = −0.19, 95% CI [−0.35, −0.03], *p* = .020). In Step 2, cognitive variables explained an additional 4.3% of the variance, for a total of 8.4%, and the overall model was significant. Verbal WM emerged as a significant positive predictor (β = 0.21, 95% CI [0.03, 0.39], *p* = .020). In Step 3, adding PWB explained an additional 7.1% of the variance (ΔR^2^ = 0.071). In the final model, verbal WM (β = 0.24, 95% CI [0.07, 0.42], *p* = .006) and PWB (β = 0.27, 95% CI [0.12, 0.42], *p* = .001) were significant positive predictors.

For VoCA-PE, the final model explained 5.6% of the variance in the old-old group (Table S4; Figure S2). In Step 1, sociodemographic variables explained 2.7% of the variance, and the model was not statistically significant, although age emerged as a significant negative predictor (β = −0.16, 95% CI [−0.32, −0.00], *p* = .048). In Step 2, sociodemographic and cognitive variables explained 5.7% of the variance, but the overall model was not significant. In Step 3, PWB did not explain a significant additional variance in the outcome (ΔR^2^ = −0.001).

## 4. Discussion

The present cross-sectional study examined the role of both some core cognitive abilities sensitive to aging-related changes—verbal WM and inhibition/interference—, and PWB in explaining essentialist and nonessentialist generalized and personal views of age-related cognitive changes (VoCA), among community-dwelling older adults.

Our results showed that verbal WM, examined with a complex span WM task, explained a significant, though modest, part of the variance in generalized VoCA. This pattern emerged for generalized nonessentialist VoCA (VoCA-GnE), whereas the overall model for generalized essentialist VoCA (VoCA-GE) was only marginally significant. These findings extend previous evidence showing that cognitive performance plays a role in VoA (Weiss & Weiss, 2016; Weiss, 2018; see also Fernández-Ballbé et al., 2023), while also suggesting that associations between verbal WM and generalized VoA apply specifically to views of age-related cognitive changes. Indeed, these findings do not concern VoA in a broad sense, but rather VoCA considered at two distinct levels and across two theoretically different belief systems about cognitive aging (essentialist and nonessentialist VoCA), with stronger associations emerging for nonessentialist views.

In particular, verbal WM performance was significantly and positively associated with generalized nonessentialist VoCA and, more cautiously, with generalized essentialist VoCA. These findings are in line with previous evidence linking verbal WM to cognitive control and metacognitive monitoring (e.g., Conte et al., 2023), suggesting that verbal WM may support the organization and “metacognitive” elaboration of beliefs about age-related cognitive change in a more general way. From a theoretical—metacognitive—perspective, object—and meta-level processes are assumed to interact through ongoing monitoring and control mechanisms (Nelson & Narens, 1994), and these processes have also been documented in older adults’ metamemory performance (Hertzog & Dunlosky, 2011). However, this interpretation should be considered in light of the different associations observed for essentialist and nonessentialist VoCA. Indeed, the relationship found was stronger for generalized nonessentialist beliefs about cognitive aging, whereas, contrary to our expectations, the association between verbal WM and generalized essentialist VoCA was significant but weak, and the overall model reached only marginal significance. One possible explanation is that generalized essentialist beliefs about cognitive aging are less closely tied to older adults’ current cognitive performance, as they may reflect more stable, socially and “culturally” shared, and internalized beliefs about age-related cognitive decline. Our interpretation is also consistent with the relatively healthy and active sample recruited for the present study: in the absence of marked cognitive difficulties, and because of their—potential—active lifestyle, older adults’ cognitive performance may be less closely related to fixed beliefs about cognitive aging, whereas more flexible and nonessentialist views may depend more directly on their perceived self-efficacy, perceived control over one’s own functioning, and the cognitive resources needed to monitor and integrate information about age-related cognitive changes as potentially malleable.

In addition, and in line with our hypotheses, as well as with evidence suggesting that older adults with greater verbal WM performance may be better able to monitor and evaluate information about their cognitive functioning and abilities (e.g., Komori, 2016), our results suggest that verbal WM performance may shape/influence beliefs about age-related cognitive changes when these are framed from a nonessentialist/malleability perspective.

Overall, these findings highlight the added value of distinguishing VoCA in terms of essentialist and nonessentialist beliefs—at both the generalized and personal level—about cognitive aging. Rather than reflecting a single and broad dimension of VoA, VoCA appeared to capture a more nuanced belief structure/system that is meaningfully related to cognitive functioning. Future studies should further examine how—core—cognitive and metacognitive mechanisms jointly contribute to VoCA across older adulthood.

Moreover, and interestingly, only resistance to PI (LST intrusion index) emerged as a significant predictor of VoCA. This finding was in line with our hypothesis and indicated that the ability to resist interference from verbal information that was previously relevant but has since become irrelevant and that is not actively removed during the complex WM span tasks (such as the LST used here) appears to be linked to nonessentialist/malleable views of age-related cognitive changes. By contrast, no such association emerged for essentialist VoCA. Thus, this specific interference mechanism seemed to be more closely related to generalized nonessentialist VoCA than generalized essentialist VoCA. One possible interpretation is that essentialist beliefs are so consolidated and socially reinforced that they are less susceptible to interference, whereas nonessentialist VoCA may require greater cognitive control to avoid reverting/rebounding to essentialist views. This interpretation is also consistent with Stereotype Embodiment Theory (B. R. Levy et al., 2018), according to which stereotypical and fixed/essentialist VoA (including those related to cognitive aging) are more socially prevalent and implicitly embedded in society (B. R. Levy, 2009), and therefore less in need of attentional control to be processed or elaborated.

Conversely, controlled cognitive inhibition (measured here as the ability to actively suppress competing dominant but irrelevant information measured with the Hayling test) was not significantly associated with either essentialist or nonessentialist generalized VoCA. Beyond confirming that inhibition is not a unitary construct but rather includes distinct mechanisms (de Ribaupierre, 2001; Borella et al., 2011), with different impacts on older adults’ cognitive functioning (Borella et al., 2008), it is possible that generalized VoCA rely more on the ability to manage interference from pre-existing stereotypical knowledge about age-related cognitive changes than on the kind of stimulus-driven control tapped by the Hayling task. In other words, the absence of an association between controlled inhibition and VoCA may reflect the specific inhibitory demands of the task: controlled inhibition may be less relevant than resistance to PI for eliciting generalized nonessentialist VoCA. It is also possible that different inhibitory mechanisms vary in their sensitivity to age-related differences in cognitive functioning, as well as in their associations with beliefs about cognitive aging.

As for personal VoCA (VoCA-PnE and VoCA-PE), verbal WM performance was found to be positively associated only with personal nonessentialist VoCA. This finding is consistent with previous studies showing that greater verbal WM is related to more positive personal VoA dimensions (e.g., Carbone et al., 2025) and extends this evidence to nonessentialist VoCA by suggesting that better verbal WM performance may support a greater sense/view of malleability in one’s own aging-related cognitive changes. By contrast, no significant association emerged with personal essentialist VoCA. This latter finding partially contrasts with evidence linking memory performance to essentialist beliefs about aging in general (Weiss, 2018) and to negative personal VoA (see Fernández-Ballbé et al., 2023). This might be because verbal WM performance, as assessed here through a complex dual-task span paradigm (Borella et al., 2008), may be more relevant for supporting flexible appraisals/evaluations of one’s own cognitive aging than for fixed beliefs about cognitive changes/decline. Indeed, personal nonessentialist VoCA may require individuals to actively monitor and update/integrate their own experiences about cognitive aging and the way they handle everyday complex task, considering age-related cognitive changes as potentially modifiable. By contrast, personal essentialist beliefs about aging-related cognitive changes may be more strongly shaped by internalized stereotypes and prior—learned—experiences of cognitive aging (in line with the Stereotype Embodiment Theory; B. R. Levy, 2009). These essentialist beliefs may therefore operate as more stable and automatic self-related beliefs, less directly dependent on the active maintenance and processing mechanisms to be used for complex task captured by—verbal—WM. This may also explain why the present findings differ from previous studies on personal VoA (e.g., Sabatini et al., 2023; Voelkner & Caskie, 2023): our results based on the presentation of a complex verbal WM task suggested that personal essentialist VoCA may not simply reflect a negative evaluation of one’s own cognitive aging, but rather a more consolidated and internalized socially-shared belief about the nature of age-related cognitive decline.

In addition, neither resistance to PI nor inhibitory control/interference performance (Hayling test) were associated with essentialist or nonessentialist personal VoCA. Personal VoCA are closely tied to self-relevant knowledge and subjective experiences of one’s own cognitive changes (Sella et al., 2026). Therefore, evaluating one’s own cognitive aging may depend less on the ability to suppress irrelevant or competing information and more on the ability to interpret/integrate, and give meaning to personally experienced cognitive changes. This may explain why inhibitory/interference mechanisms, which are mainly involved in suppressing irrelevant information or limiting their re-activation (Hasher et al., 2007; Borella et al., 2011), were not significantly associated with either personal nonessentialist or personal essentialist VoCA, and may represent cognitive processes that individuals are not explicitly aware of engaging when evaluating their own cognitive aging. At the personal level, beliefs about one’s own cognitive aging may be shaped less by the ability to resolve semantic conflicts and more by how individuals emotionally/psychologically interpret and make sense of their own cognitive experiences related to aging. Therefore, more “warmer” psychological resources—such as psychological resilience (see Sella et al., 2025)—might come into play in personal VoCA than cognitive-control mechanisms alone. Future studies should further clarify whether personal essentialist and nonessentialist VoCA are more strongly related to self-referential, affective, and metacognitive processes than to inhibitory control per se.

Our results also showed that, alongside cognitive abilities, PWB explained a significant additional part of variance in our regression models for nonessentialist VoCA, but not for essentialist ones. In particular, and in line with our expectations, greater PWB was associated with stronger generalized and personal nonessentialist VoCA, suggesting that older adults who reported higher PWB also perceived age-related cognitive changes as more malleable, both at generalized and personal levels. By contrast, PWB was not significantly associated with generalized or personal essentialist VoCA. These results suggest that PWB may be more closely related to flexible and malleable views of age-related cognitive changes than to beliefs framing cognitive aging as fixed and inevitable. This pattern may be explained by the fact that PWB reflects a eudaimonic perspective on well-being (Ryff, 2013), encompassing self-acceptance, and personal growth, which has been associated with more adaptive appraisals of aging and positive health-related outcomes in older adults (Carstensen, 2021), including cognitive functioning. Older adults with higher PWB may therefore be more likely to view cognitive aging as a process that can still be influenced through adaptation and personal agency. In contrast, essentialist beliefs about cognitive aging may be less closely tied to current PWB and may instead reflect more stable and internalized beliefs/perceptions about age-related cognitive changes as a fixed and inevitable process. Thus, PWB may be particularly relevant for supporting nonessentialist VoCA, both at the generalized and personal levels, rather than for essentialist beliefs.

Interestingly, the same pattern of associations between cognitive and psychological variables and both generalized nonessentialist (VoCA-GnE) and personal nonessentialist VoCA (VoCA-PnE) emerged only when old-old adults (75+). Given that objective age-related changes in cognitive functioning become more salient/relevant in old-old age (Craik & Salthouse, 2011), it is plausible that older adults aged 75 and over are more aware of age-related cognitive changes and that, in turn, verbal WM and resistance to PI play a greater role in shaping their nonessentialist VoCA. Moreover, resistance to PI may be particularly relevant for generalized nonessentialist VoCA in the oldest age group, as it may support the filtering of stereotypical and fixed VoA and the integration of multiple sources of verbal information into broader representations of cognitive aging. Among older adults over 75, this may reflect the fact that old age stereotype internalization tends to increase as aging becomes more self-relevant (Weiss & Kornadt, 2018; B. R. Levy, 2009), making old-old adults more exposed to and influenced by stereotyped views of age-related cognitive changes with aging. Therefore, cognitive resources may be particularly important for maintaining flexible and nonessentialist VoCA rather than relying on more fixed VoCA. Looking at the regression models, beta coefficients were generally larger in old-old adults than in the full sample (see Supplementary Materials), particularly for verbal WM and resistance to PI in relation to generalized nonessentialist VoCA and personal VoCA, whereas the association with generalized essentialist VoCA was largely comparable across groups. Overall, this pattern suggests that age stereotype internalization may increase as aging becomes more self-relevant, making old-old adults more susceptible to essentialist VoCA and, at the same time, more dependent on cognitive resources when maintaining flexible and nonessentialist VoCA.

Controlled cognitive inhibition (Hayling test) was again not significantly associated with either essentialist or nonessentialist generalized VoCA in either age group, confirming the pattern observed in the full sample. This further suggests a differentiated role of inhibitory processes, in which only resistance to PI emerged as relevant for the maintenance of generalized nonessentialist VoCA, both in the full sample and among old-old adults, with a larger beta coefficient in the latter group. Such inhibitory mechanisms may differ in their sensitivity to age-related differences in cognitive functioning (Borella et al., 2011), and in their associations with VoCA; therefore, these associations warrant further investigation.

Similarly, PWB explained a significant additional part of variance in generalized and personal nonessentialist VoCA among older adults over 75. This is consistent with previous evidence showing that, with advancing age, prioritizing positive emotions and well-being is associated with better health outcomes (Carstensen, 2021), and extends this pattern to more flexible, nonessentialist VoCA in old-old adults. Therefore, in the oldest age group (75+), PWB may represent an important psychological resource for viewing cognitive aging as malleable rather than fixed.

Overall, our analyses revealed both shared and age-specific patterns, indicating that verbal WM, resistance to PI, inhibitory mechanisms, and PWB may play partly different roles in shaping VoCA and provide further understanding of individual differences in older adulthood.

Regarding sociodemographic factors, chronological age was the only covariate negatively associated with generalized nonessentialist VoCA in young-old adults and in the full sample. This result extends previous evidence on the association between chronological age and the essentialism of aging (Weiss, 2018), that is malleable and nonessentialist beliefs about cognitive aging may become less salient with aging. These findings may indicate that chronological age is a salient factor in shaping/influencing generalized nonessentialist VoCA in young-old adults, as individuals in this life stage may begin to internalize age stereotypes (becoming self-relevant with becoming older, according to the Stereotype Embodiment Theory; B. R. Levy, 2009) and the social changes associated with aging (such as retirement and family transitions), making chronological age more closely associated with stereotyped views of cognitive decline. Conversely, in old-old adults, where individual and age-related differences as well as personal experiences may become more heterogeneous and prominent (Craik & Salthouse, 2011), cognitive and psychological factors may become more relevant than chronological age itself. Education was not a significant predictor, while gender emerged as significant covariate only in the 64–74 age group, where being female predicted lower personal essentialist VoCA; this may suggest that women, compared with men, perceive less agency and fewer possibilities for change in relation to their own cognitive aging. This interpretation is consistent with the “double standard of aging” (Sontag, 1978) and previous evidence according to which women may experience their “older” age more negatively because of the combined effects of gender and chronological age (Ishikawa, 2023), extending this pattern to perceptions of their own age-related cognitive changes as well. Interestingly, this gender difference in VoCA did not emerge among old-old adults. It is possible that, in very old age, as the impact of aging on cognitive functioning becomes more salient (Craik & Salthouse, 2011), cognitive abilities and PWB weigh more strongly than gender (as found here), and in line with the Stereotype Embodiment Theory (B. R. Levy et al., 2018), negative age-related views in adults over 75 may therefore apply regardless of gender. In general, these findings are consistent with Sella et al. (2026) and indicate that sociodemographic factors may have limited relevance for shaping/influencing VoCA in older adulthood.

In addition, some relevant findings emerging from the correlations among the VoCA dimensions should also be considered when interpreting these results (Table 2). Our results showed strong associations among essentialist and nonessentialist VoCA dimensions (in the full sample and within both old age groups), and these correlations were stronger than those reported in the original validation study with a mixed sample of middle-aged and older adults (Sella et al., 2026). This partially different pattern of results from Sella et al. (2026) suggests that, in older adulthood, the different VoCA dimensions may partly overlap and share a broader component related to the salience of beliefs about age-related cognitive changes, possibly reflecting the greater relevance of such changes with aging. However, these associations do not imply that essentialist and nonessentialist VoCA are redundant constructs. This pattern of associations is consistent with broader multidimensional approaches to VoA, which distinguish different belief orientations within the same belief system, such as essentialist and nonessentialist beliefs about generalized views of aging (Weiss & Weiss, 2016), or perceived age-related gains and losses within the awareness of age-related change framework for personal VoA (Sabatini et al., 2023), and extends this multidimensional perspective to VoCA. In addition, our results suggest that essentialist and nonessentialist VoCA should not be interpreted simply as opposite ends of a single continuum.

Despite these interesting findings, some limitations should be acknowledged. First, due to the cross-sectional design, the direction of the associations between VoCA, cognition and PwB cannot be determined. Although the present regression models examined cognitive functioning and PWB as predictors of VoCA, the reverse direction is also plausible: essentialist and nonessentialist VoCA may influence cognitive performance, consistent with previous evidence showing that personal VoA dimensions can act as antecedents/predictors of cognitive functioning (e.g., Fernández-Ballbé et al., 2023; Carbone et al., 2025), as well as psychological health and quality of life (Sella et al., 2025; Carbone et al., 2026). Longitudinal studies and more advanced statistical approaches (such as structural equation modeling), applied to larger samples, also considering socioeconomic characteristics (e.g., socioeconomic status) of the sample could provide deeper insights into the temporal or causal direction or the bidirectional relationships among cognitive, psychological, and VoCA dimensions. Moreover, the regression models, whether statistically significant or marginally significant, explained a modest part of variance in VoCA, generally below one fifth (Table 2 and Table 3; and Supplementary Materials), indicating that verbal WM, inhibition/interference processes, and PWB capture only part of the individual differences in essentialist and nonessentialist VoCA.

Second, although the overall validity of the VoCA was generally good, in line with Sella et al. (2026), the internal consistency of the VoCA-PnE subscale was marginally acceptable in the present sample. On the one hand, this may partly reflect the small number of items included in the VoCA-PnE subscale, given that omega estimates are influenced by the number of items (e.g., Edwards et al., 2021); on the other hand, it may also reflect the more nuanced nature of personal nonessentialist views of cognitive aging, which involve evaluations of the malleability and modifiability of one’s own cognitive aging only partially captured by the current VoCA-PnE items. Personal nonessentialist VoCA may, to some extent, entail personal appraisals of control and action over one’s own cognitive functioning, potentially involving aspects related to self-efficacy (Bandura & Wessels, 1997) and internal control beliefs (Lachman, 2006), which may only be partially captured by the current VoCA-PnE items; therefore, findings involving this subscale should be interpreted with caution and further examined in future studies.

A further limitation is that these findings refer specifically to verbal WM, as assessed by the LST, and should not be generalized to WM as a whole. Future studies should thus replicate our results including visuospatial complex WM measures to clarify whether essentialist and nonessentialist VoCA relationship with WM vary depending on the nature of the stimuli presented (verbal vs. visuospatial).

Another limitation concerns the fixed order of the measures administration. This order was chosen to vary the cognitive load required, alternating most cognitively demandingtask—such as the LST—to less demanding ones (not included in the present analyses).

This choice was intended to maximize completion rates and reduce fatigue during the most demanding part of the assessment. However, because the order of cognitive tasks and questionnaires was not counterbalanced, potential practice and/or fatigue effects cannot be ruled out; future studies should counterbalance task order to better control for order-related effects.

Finally, including broader VoA measures alongside VoCA would also help clarify whether they overlap or explain unique variance in relation to cognitive domains and psychological factors. Finally, given that subjective cognitive concerns may already emerge in midlife, future studies should include younger adults and individuals at risk of cognitive difficulties. This would help clarify the role of cognitive abilities and PWB in VoCA across both healthy and pathological aging.

## 5. Conclusions

In conclusion, our findings suggest that core cognitive mechanisms in aging—verbal WM and cognitive inhibition, particularly resistance to PI—, and PWB are associated with how older adults perceive aging-related cognitive changes. These associations, however, varied depending on the type of VoCA considered—generalized and personal—, the approach to age-related cognitive changes—essentialist and nonessentialist—, and the age group examined. Verbal WM and PWB were mainly associated with stronger generalized and personal nonessentialist VoCA. Cognitive inhibition, specifically proactive interference resistance, was associated only with generalized nonessentialist VoCA. These associations were particularly evident in old-old adults.

Overall, these cross-sectional findings contribute to a better understanding of cognitive resources, including verbal WM and related inhibitory processes, as well as PWB, as potential antecedents of VoCA. From an applied standpoint, these findings suggest that assessing VoCA alongside cognitive functioning and PWB may be particularly useful in old-old adults, for whom the associations with nonessentialist VoCA were more evident. Evidence-based psychoeducational and metacognitive interventions (e.g., Sella et al., 2023 for a review), tailored to different older-age groups as done here, could focus on promoting more flexible views of age-related cognitive changes with aging, reinforcing perceived cognitive malleability, personal agency and self-efficacy, and supporting active cognitive engagement and psychological resources to promote PWB as part of new and individualized healthy aging programs.

## Figures and Tables

**Figure 1:**
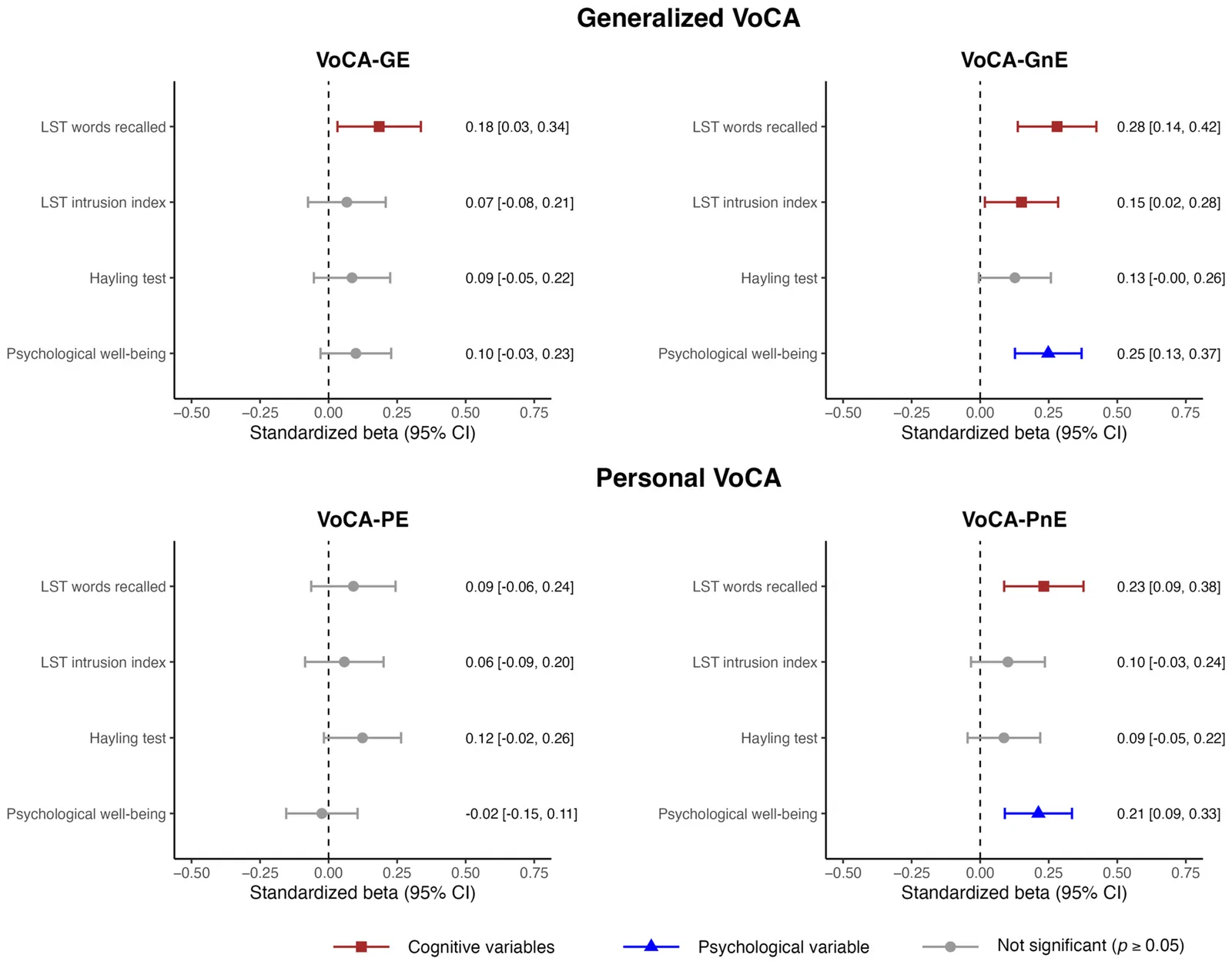
Standardized regression coefficients (95% CIs) for cognitive and psychological predictors of generalized and personal VoCA in older adults. Models were adjusted for age, gender, and education.

**Table 1:** Descriptive statistics for study variables in the total sample, young-old, and old-old groups.

	Total Sample (*N* = 237; 64–85 Years)	Young-Old Adults (*N* = 83; 64–74 Years)	Old-Old Adults (*N* = 154; 75–85 Years)
	M (SD)	M (SD)	M (SD)
Age	76.76 (5.66)	70.24 (3.23)	80.27 (2.88)
Female, *N* (%)	137 (57.81%)	51 (61.45%)	86 (55.84%)
Education	11.50 (4.29)	12.66 (4.43)	10.88 (4.09)
Retirement, *N* (%)	224 (94.5%)	74 (89.2%)	150 (97.4%)
VoCA-GnE	14.19 (4.97)	15.01 (3.76)	13.74 (5.47)
VoCA-GE	11.32 (4.13)	11.67 (3.20)	11.13 (4.55)
VoCA-PnE	11.65 (3.96)	12.66 (2.73)	11.11 (4.39)
VoCA-PE	15.37 (6.27)	15.58 (5.18)	15.25 (6.80)
LST words recalled	20.39 (4.59)	21.98 (4.50)	19.46 (4.39)
LST intrusion index	0.18 (0.17)	0.14 (0.13)	0.20 (0.19)
Hayling test	6.27 (2.24)	7.34 (2.21)	5.69 (2.04)
PWB	114.08 (12.57)	111.94 (13.55)	115.24 (11.88)

Note. VoCA = Views of Cognitive Aging; GnE = Generalized nonessentialism; GE = Generalized essentialism; PnE = Personal nonessentialism; PE = Personal essentialism; LST = Listening Span test; PWB = Psychological Well-Being.

**Table 4:** Hierarchical Regression Analyses Predicting Personal Nonessentialist and Essentialist VoCA (-PnE and -PE) in the Full Sample.

	VoCA-PnE									VoCA-PE								
	*Step 1*			*Step 2*			*Step 3*			*Step 1*			*Step 2*			*Step 3*		
	*β*	*95% CI*	*p*	*β*	*95% CI*	*p*	*β*	*95% CI*	*p*	*β*	*95% CI*	*p*	*β*	*95% CI*	*p*	*β*	*95% CI*	*p*
Intercept	−0.00	−0.12–0.12	<.001	0.00	−0.12–0.12	<.001	−0.00	−0.12–0.12	.048	−0.00	−0.13–0.13	<.001	−0.00	−0.13–0.13	.042	0.00	−0.13–0.13	.074
Age	−0.24	−0.37–−0.11	<.001	−0.18	−0.32–−0.05	.008	−0.18	−0.32–−0.05	.007	−0.06	−0.19–0.07	.357	−0.02	−0.16–0.12	.801	0.00	−0.14–0.14	.985
Gender	−0.04	−0.16–0.09	.573	−0.06	−0.19–0.07	.360	−0.04	−0.17–0.08	.518	−0.11	−0.24–0.02	.083	−0.13	−0.27–−0.00	.047	−0.13	−0.26–0.00	.057
Education	0.04	−0.09–0.17	.538	−0.03	−0.17–0.11	.703	−0.04	−0.18–0.10	.562	0.04	−0.10–0.17	.600	−0.01	−0.15–0.14	.935	−0.02	−0.16–0.13	.815
LST words recalled	-	-	-	0.22	0.07–0.37	.003	0.23	0.09–0.38	.002	-	-	-	0.07	−0.08–0.22	.359	0.09	−0.06–0.24	.248
LST intrusion index	-	-	-	0.09	−0.05–0.23	.188	0.10	−0.03–0.24	.140	-	-	-	0.06	−0.09–0.20	.442	0.06	−0.09–0.20	.430
Hayling test	-	-	-	0.06	−0.08–0.19	.392	0.09	−0.05–0.22	.199	-	-	-	0.13	−0.01–0.27	.073	0.12	−0.02–0.26	.085
PWB	-	-	-	-	-	-	0.21	0.09–0.33	.001	-	-	-	-	-	-	−0.02	−0.15–0.11	.711
R^2^/adjusted R^2^	0.065/0.053			0.105/0.081			0.149/0.122			0.020/0.007			0.039/0.014			0.038/0.008		
ΔR^2^/adjusted R^2^	-			0.040/0.028			0.044/0.041			-			0.019/0.007			−0.001/−0.006		
F	5.398			4.481			5.686			1.571			1.558			1.287		
*p*	.001			<.001			<.001			.197			.161			.257		

Note. VoCA = Views of Cognitive Aging; PnE = Personal nonessentialism; PE = Personal essentialism; LST = Listening Span test; PWB = Psychological Well-Being.

## Data Availability

The data that support the findings of this study are available on request from the corresponding author. The data are not publicly available due to the confidential nature of the dataset collected.

## Supplementary Materials

The following supporting information can be downloaded at https://www.mdpi.com/article/10.3390/jintelligence14100238/s1, Table S1. Hierarchical regression analyses predicting generalized nonessentialist and essentialist VoCA (-GnE and -GE) in the young-old group; Table S2. Hierarchical regression analyses predicting personal nonessentialist and essentialist VoCA (-PnE and -PE) in the young-old group; Table S3. Hierarchical regression analyses predicting generalized nonessentialist and essentialist VoCA (-GnE and -GE) in the old-old group; Table S4. Hierarchical regression analyses predicting personal nonessentialist and essentialist VoCA (-PnE and -PE) in the old-old group; Figure S1. Standardized regression coefficients (95% CIs) for cognitive and psychological predictors of generalized and personal VoCA in older adults aged 64–74 years old. Models were adjusted for age, gender, and education; Figure S2. Standardized regression coefficients (95% CIs) for cognitive and psychological predictors of generalized and personal VoCA in older adults aged 75–85 years old. Models were adjusted for age, gender, and education.

## Abbreviations

The following abbreviations are used in this manuscript:

AARC	Awareness Of Age-Related Changes
ATOA	Attitudes Toward one’s Own Aging
BEN-SSC	Questionario Ben-Essere Psicologico
GE	Generalized Essentialism
GnE	Generalized Nonessentialism
LST	Listening Span Test
PWB	Psychological Well-Being
PI	Proactive Interference
PnE	Personal Nonessentialism
PE	Personal Essentialism
VoA	Views of Aging
VoCA	Views of Cognitive Aging
WM	Working Memory

## References

[B1-jintelligence-14-00238] Bandura A., Wessels S. (1997). Self-efficacy.

[B2-jintelligence-14-00238] Benjamini Y., Hochberg Y. (1995). Controlling the false discovery rate: A practical and powerful approach to multiple testing. Journal of the Royal Statistical Society: Series B (Methodological).

[B3-jintelligence-14-00238] Borella E., Carretti B., De Beni R. (2008). Working memory and inhibition across the adult life-span. Acta Psychologica.

[B4-jintelligence-14-00238] Borella E., Ludwig C., Fagot D., De Ribaupierre A. (2011). The effect of age and individual differences in attentional control: A sample case using the Hayling test. Archives of Gerontology and Geriatrics.

[B5-jintelligence-14-00238] Boylan J. M., Choi J. H., Kirsch J. A., Ryff C. D. (2025). Educational disparities in 20-year trajectories of psychological well-being in a national sample. Scientific Reports.

[B6-jintelligence-14-00238] Boylan J. M., Ryff C. D. (2015). Psychological well-being and metabolic syndrome: Findings from the midlife in the United States national sample. Biopsychosocial Science and Medicine.

[B7-jintelligence-14-00238] Brothers A., Miche M., Wahl H.-W., Diehl M. (2017). Examination of associations among three distinct subjective aging constructs and their relevance for predicting developmental correlates. The Journals of Gerontology: Series B, Psychological Sciences and Social Sciences.

[B8-jintelligence-14-00238] Carbone E., Paccagnella O., Borella E. (2025). Gauging the link between different views of aging facets and their associations with cognitive and mood functioning in midlife and older age: A cross-sectional study. Frontiers in Psychology.

[B9-jintelligence-14-00238] Carbone E., Sella E., Ghisletta P., Borella E. (2026). Personal views of aging and quality of life in midlife and older age: The role of cognitive reserve. Frontiers in Psychology.

[B10-jintelligence-14-00238] Carstensen L. L. (2021). Socioemotional selectivity theory: The role of perceived endings in human motivation. The Gerontologist.

[B11-jintelligence-14-00238] Conte N., Fairfield B., Padulo C., Pelegrina S. (2023). Metacognition in working memory: Confidence judgments during an n-back task. Consciousness and Cognition.

[B12-jintelligence-14-00238] Craik F. I. M., Salthouse T. A. (2011). The handbook of aging and cognition.

[B13-jintelligence-14-00238] Dapor C., Devita M., Iannizzi P., Arbia E., Bruzzano A., Dessì M., Lupi D., Rolandino G. M., Rossi M., Saccomano A., Siccardi E., Simonetto A., Vuerich G., Zuliani S., Priftis K. (2025). The Montreal cognitive assessment (MoCA) 8.1 version, including the memory index score (MoCA-MIS): Italian norms. Neurological Sciences.

[B14-jintelligence-14-00238] De Beni R., Borella E., Carretti B., Marigo C., Nava L. A. (2008). BAC. Portfolio per la valutazione del benessere e delle abilità cognitive nell’età adulta e avanzata *[The assesment of well-being and cognitive abilities in adulthood and aging]*.

[B15-jintelligence-14-00238] de Ribaupierre A. (2001). Working memory and attentional processes across the lifespan. Lifespan development of human memory.

[B16-jintelligence-14-00238] Diehl M., Rebok G. W., Roth D. L., Nehrkorn-Bailey A., Rodriguez D., Tseng H. Y., Chen D. (2023). Examining the malleability of negative views of aging, self-efficacy beliefs, and behavioral intentions in middle-aged and older adults. The Journals of Gerontology: Series B.

[B17-jintelligence-14-00238] Diehl M., Wahl H.-W., Barrett A. E., Brothers A. F., Miche M., Montepare J. M., Westerhof G. J., Wurm S. (2014). Awareness of aging: Theoretical considerations on an emerging concept. Developmental Review.

[B18-jintelligence-14-00238] Edwards A. A., Joyner K. J., Schatschneider C. (2021). A simulation study on the performance of different reliability estimation methods. Educational and Psychological Measurement.

[B19-jintelligence-14-00238] Fernández-Ballbé Ó., Martin-Moratinos M., Saiz J., Gallardo-Peralta L., Barrón López de Roda A. (2023). The relationship between subjective aging and cognition in elderly people: A systematic review. Healthcare.

[B20-jintelligence-14-00238] Folstein M. F., Folstein S. E., McHugh P. R. (1975). “Mini-mental state”: A practical method for grading the cognitive state of patients for the clinician. Journal of Psychiatric Research.

[B21-jintelligence-14-00238] Hajek A., Buczak-Stec E., Riedel-Heller S. G., König H. H. (2020). Reductions in cognitive functioning are associated with decreases in satisfaction with aging. Longitudinal findings based on a nationally representative sample. Archives of Gerontology and Geriatrics.

[B22-jintelligence-14-00238] Hasher L., Lustig C., Zacks R. T. (2007). Inhibitory mechanisms and the control of attention. Variation in working memory.

[B23-jintelligence-14-00238] Hertzog C., Dunlosky J. (2011). Metacognition in later adulthood: Spared monitoring can benefit older adults’ self-regulation. Current Directions in Psychological Science.

[B24-jintelligence-14-00238] Ishikawa M. (2023). Internalization of negative societal views on old age into self-perceptions of aging: Exploring factors associated with self-directed ageism. Frontiers in Sociology.

[B25-jintelligence-14-00238] Komori M. (2016). Effects of working memory capacity on metacognitive monitoring: A study of group differences using a listening span test. Frontiers in Psychology.

[B26-jintelligence-14-00238] Lachman M. E. (2006). Perceived control over aging-related declines: Adaptive beliefs and behaviors. Current Directions in Psychological Science.

[B27-jintelligence-14-00238] Lachman M. E., Baltes P., Nesselroade J. R., Willis S. L. (1982). Examination of personality-ability relationships in the elderly: The role of the contextual (interface) assessment mode. Journal of Research in Personality.

[B28-jintelligence-14-00238] Lawton M. P. (1975). The Philadelphia geriatric center morale scale: A revision. Journal of Gerontology.

[B29-jintelligence-14-00238] Levy B. (1996). Improving memory in old age through implicit self-stereotyping. Journal of Personality and Social Psychology.

[B30-jintelligence-14-00238] Levy B. R. (2009). Stereotype embodiment: A psychosocial approach to aging. Current Directions in Psychological Science.

[B31-jintelligence-14-00238] Levy B. R., Slade M. D., Pietrzak R. H., Ferrucci L. (2018). Positive age beliefs protect against dementia even among elders with high-risk gene. PLoS ONE.

[B32-jintelligence-14-00238] Mei S., Zheng C., Liang L., Kiyum M., Fei J., Yuan T., Wang H., Gao J., Lin X. (2026). Trajectories of cognitive ability and attitudes toward own aging in older adults: A conditional parallel latent growth model. BMC Geriatrics.

[B33-jintelligence-14-00238] Montepare J. M. (2009). Subjective age. International Journal of Behavioral Development.

[B34-jintelligence-14-00238] Nelson T. O., Narens L. (1994). Why investigate metacognition?. Metacognition: Knowing about knowing.

[B35-jintelligence-14-00238] Nosrati P., Momeni K., Wahl H.-W. (2026). Examining the relationship between self-perceptions of aging, psychological well-being and health-promoting lifestyle: A cross-sectional study in Iran. Journal of Cross-Cultural Gerontology.

[B36-jintelligence-14-00238] Palombo M. O., Foran A. M. (2025). Effectiveness of the Hayling and Brixton tests for detecting dementia, progressive cognitive decline, and mild cognitive impairment in middle to older aged adults: A systematic review and meta-analysis. Neuropsychology Review.

[B37-jintelligence-14-00238] Pliatsikas C., Veríssimo J., Babcock L., Pullman M. Y., Glei D. A., Weinstein M., Goldman N., Ullman M. T. (2019). Working memory in older adults declines with age, but is modulated by sex and education. Quarterly Journal of Experimental Psychology.

[B38-jintelligence-14-00238] Prati G. (2024). Changes in essentialist beliefs about cognitive aging predicts changes in mental health: Evidence from a 10-year longitudinal study. Psychology and Aging.

[B39-jintelligence-14-00238] R Core Team (2026). R: A language and environment for statistical computing *[Computer software]. R Foundation for Statistical Computing*.

[B40-jintelligence-14-00238] Ryff C. D. (2013). Psychological well-being revisited: Advances in the science and practice of eudaimonia. Psychotherapy and Psychosomatics.

[B41-jintelligence-14-00238] Ryff C. D., Keyes C. L. M. (1995). The structure of psychological well-being revisited. Journal of Personality and Social Psychology.

[B42-jintelligence-14-00238] Sabatini S., Ukoumunne O. C., Ballard C., Collins R., Anstey K. J., Diehl M., Brothers A., Wahl H.-W., Corbett A., Hampshire A., Brooker H., Clare L. (2021). Cross-sectional association between objective cognitive performance. International Psychogeriatrics.

[B43-jintelligence-14-00238] Sabatini S., Wahl H.-W., Diehl M., Clare L., Ballard C., Brooker H., Corbett A., Hampshire A., Stephan B. C. M. (2023). Testing bidirectionality. The Journals of Gerontology: Series B.

[B44-jintelligence-14-00238] Schönstein A., Dallmeier D., Denkinger M., Rothenbacher D., Klenk J., Bahrmann A., Wahl H. W. (2021). Health and subjective views on aging: Longitudinal findings from the ActiFE Ulm study. The Journals of Gerontology: Series B.

[B45-jintelligence-14-00238] Sella E., Carbone E., Borella E. (2025). Associations between personal views of aging and quality of life in midlife and older age: The mediating role of psychological resilience. Healthcare.

[B46-jintelligence-14-00238] Sella E., Carbone E., Ghisletta P., Borella E. (2026). Views of cognitive aging in midlife and older age: Development of a new scale. Frontiers in Psychology.

[B47-jintelligence-14-00238] Sella E., Carbone E., Vincenzi M., Toffalini E., Borella E. (2023). Efficacy of memory training interventions targeting metacognition for older adults: A systematic review and meta-analysis. Aging & Mental Health.

[B48-jintelligence-14-00238] Shrira A., Palgi Y., Diehl M., Diehl M., Wahl H.-W. (2022). Advancing the field of subjective views of aging. Subjective views of aging.

[B49-jintelligence-14-00238] Siegel A. L., Eich T. S. (2021). Age, sex, and inhibitory control: Identifying a specific impairment in memorial, but not perceptual, inhibition in older women. The Journals of Gerontology: Series B.

[B50-jintelligence-14-00238] Sontag S., Carver V., Liddiard P. (1978). The double standard of ageing. An ageing population.

[B51-jintelligence-14-00238] Voelkner A. R., Caskie G. I. (2023). Awareness of age-related change. Aging, Neuropsychology, and Cognition.

[B52-jintelligence-14-00238] Wang G., Shi J., Yao J., Fu H. (2020). Relationship between activities of daily living and attitude toward own aging among the elderly in China: A chain mediating model. The International Journal of Aging and Human Development.

[B53-jintelligence-14-00238] Weiss D. (2018). On the inevitability of aging: Essentialist beliefs moderate the impact of negative age stereotypes on older adults’ memory performance and physiological reactivity. The Journals of Gerontology: Series B.

[B54-jintelligence-14-00238] Weiss D., Diehl M. (2021). Measuring (non) essentialist beliefs about the process of aging. The Journals of Gerontology: Series B.

[B55-jintelligence-14-00238] Weiss D., Kornadt A. E. (2018). Age-stereotype internalization and dissociation: Contradictory processes or two sides of the same coin?. Current Directions in Psychological Science.

[B56-jintelligence-14-00238] Weiss D., Weiss M. (2016). The interplay of subjective social status and essentialist beliefs about cognitive aging on cortisol reactivity to challenge in older adults. Psychophysiology.

[B57-jintelligence-14-00238] Westerhof G. J., Nehrkorn-Bailey A. M., Tseng H.-Y., Brothers A., Siebert J. S., Wurm S., Wahl H.-W., Diehl M. (2023). Longitudinal effects of subjective aging. Psychology and Aging.

