# Supplementary material for: Essentialist and Nonessentialist Views of Cognitive Aging in Older Age: The Role of Working Memory, Cognitive Inhibition and Psychological Well-Being
Source: J Intell. 2026 Oct 2;14(10):238. doi: 10.3390/jintelligence14100238 (PMC13705725; doi:10.3390/jintelligence14100238)
Supplement: Supplementary file 1 [file jintelligence-14-00238-s001.zip › jintelligence-4448470-supplementary.pdf]

Supplementary Material

Essentialist and Nonessentialist Views of Cognitive Aging in Older Age: The Role of Working Memory, Cognitive Inhibition and Psychological Well-Being

PART S1 – Additional Tables of hierarchical regression analyses
PART S2 - Additional Figures of regression models

PART S1 – Additional Tables of hierarchical regression analyses

1.1 Hierarchical regression analyses in young-old adults (64-74 years)

Table S1. Hierarchical Regression Analyses Predicting Generalized Nonessentialist and Essentialist VoCA (-GnE and -GE) in the young-old group.

	VOCA-GnE									VOCA-GE								
	Step 1			*Step 2*			*Step 3*			*Step 1*			*Step 2*			*Step 3*		
	β	*95% CI*	*p*	*β*	*95% CI*	*p*	*β*	*95% CI*	*p*	*β*	*95% CI*	*p*	*β*	*95% CI*	*p*	*β*	*95% CI*	*p*
Intercept	.00	-.22 - .22	.369	.00	-.22 - .22	.798	.00	-.22 - .22	.981	.00	-.21 - .21	.446	.00	-.21 - .21	.662	.00	-.21 - .21	.898
Age	.07	-.15 - .30	.527	.09	-.13 - .31	.432	.06	-.16 - .29	.571	.09	-.13 - .30	.436	.09	-.13 - .31	.403	.07	-.15 - .29	.551
Gender	-.02	-.24 - .21	.873	-.07	-.29 - .16	.558	-.08	-.30 - .15	.483	-.19	-.40 - .03	.091	-.20	-.43 - .02	.073	-.22	-.44 - .00	.054
Education	-.02	-.24 - .21	.873	-.04	-.30 - .22	.776	-.06	-.32 - .20	.664	.18	-.04 - .40	.106	.08	-.18 - .34	.539	.06	-.20 - .31	.650
LST words recalled				.19	-.07 - .46	.156	.17	-.10 - .43	.220				.15	-.12 - .41	.269	.12	-.14 - .38	.371
LST intrusion index				.16	-.08 - .40	.183	.13	-.11 - .37	.290				-.03	-.27 - .21	.806	-.06	-.30 - .17	.589
Hayling test				.18	-.05 - .41	.130	.21	-.02 - .45	.074				.07	-.16 - .30	.552	.11	-.12 - .34	.360
PWB				-	-	-	.17	-.05 - .40	.132				-	-	-	.19	-.03 - .41	.096
R^2 / adjusted R^2		.009 / -.029			.083 / .011			.111 / .028			.081 / .046			.106 / .036			.139 / .059	
ΔR^2 / adjusted R^2		-			.074 / .040			.028 / .017			-			.025 / −.010			.033 / .023	
F		.2376			1.149			1.334			2.328			1.509			1.731	
p		.870			.342			.247			.081			.187			.115	

Note. VoCA = Views of Cognitive Aging; GnE = Generalized nonessentialism; GE = Generalized essentialism; LST = Listening Span test; PWB = Psychological Well-Being.

Table S2. Hierarchical Regression Analyses Predicting Personal Nonessentialist and Essentialist VoCA (-PnE and -PE) in the young-old group.

	**VOCA-PnE**									**VOCA-PE**								
	Step 1			*Step 2*			*Step 3*			*Step 1*			*Step 2*			*Step 3*		
	β	*95% CI*	*p*	*β*	*95% CI*	*p*	*β*	*95% CI*	*p*	*β*	*95% CI*	*p*	*β*	*95% CI*	*p*	*β*	*95% CI*	*p*
Intercept	.00	-.22 - .22	**.002**	.00	-.22 - .22	.023	.00	-.22 - .22	.038	-.00	-.21 - .21	.443	-.00	-.21 - .21	.348	-.00	-.21 - .21	.365
Age	-.14	-.36 - .08	.216	-.11	-.33 - .11	.321	-.13	-.35 - .10	.265	.08	-.13 - .29	.442	.06	-.15 - .28	.549	.06	-.15 - .28	.561
Gender	-.13	-.35 - .10	.263	-.15	-.37 - .07	.186	-.16	-.38 - .07	.166	-.31	-.52 - -.10	**.004**	-.34	-.55 - -.12	.003	-.34	-.55 - -.12	.003
Education	.02	-.20 - .24	.875	-.06	-.32 - .20	.645	-.07	-.33 - .19	.582	.14	-.07 - .35	.175	.15	-.09 - .40	.223	.15	-.10 - .40	.230
LST words recalled				.25	-.02 - .51	.064	.23	-.03 - .50	.086				-.12	-.38 - .13	.336	-.12	-.38 - .13	.339
LST intrusion index				.19	-.04 - .43	.108	.17	-.07 - .42	.156				-.03	-.26 - .20	.808	-.03	-.26 - .21	.805
Hayling test				.04	-.20 - .27	.759	.06	-.18 - .29	.629				.16	-.06 - .38	.159	.16	-.07 - .39	.167
PWB				-	-	-	.11	-.12 - .33	.349				-	-	-	.01	-.21 - .23	.955
R^2 / adjusted R^2		.034 / -.003			.095 / .023			.105 / .022			.137 / .104			.166 / .100			.166 / .088	
Δ R^2 / adjusted R^2					.061 / .026			.010 / -.001						.029 / −.004			.000 / -.012	
F		.9151			1.324			1.260			4.171			2.518			2.131	
p		.438			.256			.282			.009			.028			.050	

Note. VoCA = Views of Cognitive Aging; PnE = Personal nonessentialism; PE = Personal essentialism; LST = Listening Span test; PWB = Psychological Well-Being.

1.2 Hierarchical regression analyses in old-old adults (75-85 years)

Table S3. Hierarchical Regression Analyses Predicting Generalized Nonessentialist and Essentialist VoCA (-GnE and -GE) in the old-old group.

	VOCA-GnE									VOCA-GE								
	Step 1			*Step 2*			*Step 3*			*Step 1*			*Step 2*			*Step 3*		
	β	*95% CI*	*p*	*β*	*95% CI*	*p*	*β*	*95% CI*	*p*	*β*	*95% CI*	*p*	*β*	*95% CI*	*p*	*β*	*95% CI*	*p*
Intercept	.00	-.16 - .16	**<.001**	.00	-.15 - .15	.014	-.00	-.14 - .14	.568	-.00	-.16 - .16	**.002**	-.00	-.16 - .16	.022	.00	-.16 - .16	.134
Age	-.22	-.38 - -.07	**.006**	-.18	-.33 - -.02	.027	-.13	-.28 - .02	.088	-.17	-.33 - -.01	**.038**	-.14	-.31 - .02	.085	-.11	-.28 - .05	.184
Gender	.03	-.13 - .20	.672	.00	-.16 - .16	.981	.04	-.11 - .20	.576	-.03	-.19 - .13	.705	-.05	-.22 - .12	.551	-.03	-.20 - .13	.689
Education	.07	-.09 - .23	.410	.01	-.15 - .18	.890	.01	-.14 - .17	.861	.03	-.14 - .19	.758	-.01	-.18 - .16	.888	-.02	-.20 - .15	.786
LST words recalled				.28	.11 - .45	.002	.31	.15 - .48	<.001				.15	-.04 - .33	.114	.17	-.01 - .36	.063
LST intrusion index				.15	-.02 - .32	.079	.19	.02 - .35	.026				.09	-.09 - .27	.324	.10	-.08 - .28	.278
Hayling test				.07	-.09 - .23	.386	.10	-.05 - .25	.204				.09	-.07 - .25	.285	.09	-.07 - .26	.264
PWB				-	-	-	.29	.14 - .44	<.001				-	-	-	.07	-.10 - .23	.414
R^2 / adjusted R^2		.057 / .038			.128 / .092			.214 / .177			.032 / .012			.058 / .020			.061 / .015	
ΔR^2 / adjusted R^2					.071 / .054			.086 / .085						.026 / .008			.003 / -.005	
F		3.022			3.586			5.656			1.632			1.513			1.339	
p		.032			<.001			<.001			.184			.178			.236	

Note. VoCA = Views of Cognitive Aging; GnE = Generalized nonessentialism; GE = Generalized essentialism; LST = Listening Span test; PWB = Psychological Well-Being.

Table S4. Hierarchical Regression Analyses Predicting Personal Nonessentialist and Essentialist VoCA (-PnE and -PE) in the old-old group.

	**VOCA-PnE**									**VOCA-PE**								
	Step 1			*Step 2*			*Step 3*			*Step 1*			*Step 2*			*Step 3*		
	β	*95% CI*	*p*	*β*	*95% CI*	*p*	*β*	*95% CI*	*p*	*β*	*95% CI*	*p*	*β*	*95% CI*	*p*	*β*	*95% CI*	*p*
Intercept	-.00	-.16 - .16	**.001**	.00	-.16 - .16	.024	.00	-.15 - .15	.601	.00	-.16 - .16	**.003**	.00	-.16 - .16	.025	-.00	-.16 - .16	.054
Age	-.19	-.35 - -.03	**.020**	-.15	-.31 - .01	.071	-.10	-.26 - .05	.193	-.16	-.32 - -.00	**.048**	-.14	-.31 - .02	.088	-.12	-.28 - .05	.160
Gender	.00	-.16 - .16	.993	-.02	-.18 - .15	.829	.02	-.14 - .18	.797	-.03	-.19 - .13	.731	-.05	-.21 - .12	.588	-.04	-.21 - .13	.631
Education	.06	-.11 - .22	.496	.00	-.17 - .17	.971	.00	-.16 - .17	.962	-.00	-.17 - .16	.961	-.04	-.21 - .13	.618	-.06	-.23 - .11	.492
LST words recalled				.21	.03 - .39	.020	.24	.07 - .42	.006				.11	-.07 - .29	.249	.13	-.05 - .31	.161
LST intrusion index				.08	-.10 - .25	.370	.11	-.06 - .28	.202				.08	-.10 - .26	.365	.08	-.10 - .26	.377
Hayling test				.08	-.08 - .24	.350	.10	-.05 - .26	.197				.14	-.03 - .30	.099	.13	-.03 - .30	.117
PWB				-	-	-	.27	.12 - .42	.001				-	-	-	-.03	-.20 - .13	.672
R^2 / adjusted R^2		.041 / .022			.084 / .046			.155 / .115			.027 / .008			.057 / .018			.056 / .010	
ΔR^2 / adjusted R^2					.043 / .024			.071 / .069						.030 / .010			-.001 / -.008	
F		2.130			2.235			3.813			1.389			1.473			1.219	
p		.099			.043			.001			.249			.191			.296	

Note. VoCA = Views of Cognitive Aging; PnE = Personal nonessentialism; PE = Personal essentialism; LST = Listening Span test; PWB = Psychological Well-Being.

PART S2 – Additional Figures of regression models

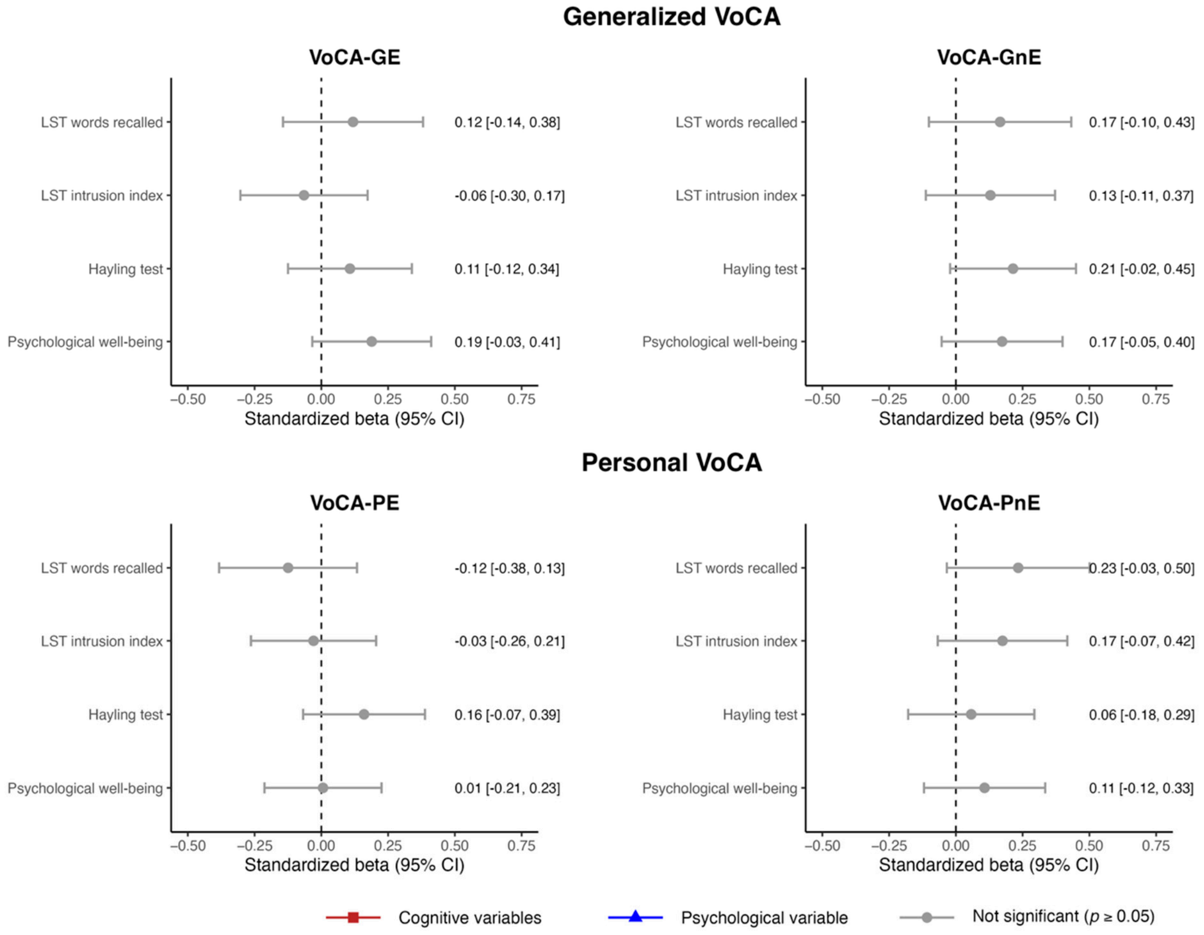

Figure S1. Standardized regression coefficients (95% CIs) for cognitive and psychological predictors of generalized and personal VoCA in older adults aged 64-74 years old. Models were adjusted for age, gender, and education.

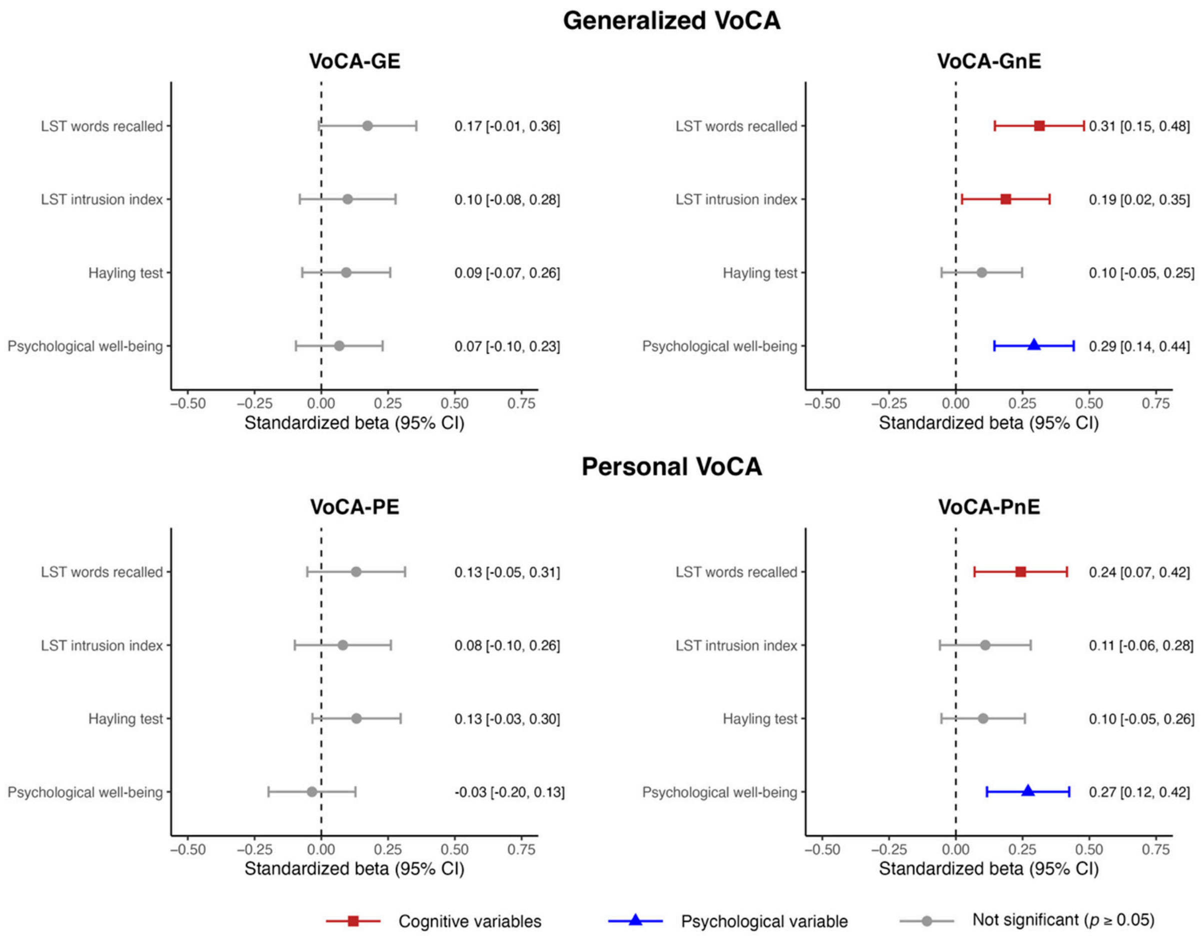

Figure S2. Standardized regression coefficients (95% CIs) for cognitive and psychological predictors of generalized and personal VoCA in older adults aged 75-85 years old. Models were adjusted for age, gender, and education.
